# Chronic disease management: a qualitative study investigating the barriers, facilitators and incentives perceived by Swiss healthcare stakeholders

**DOI:** 10.1186/1472-6963-12-176

**Published:** 2012-06-22

**Authors:** Stéphanie Lauvergeon, Bernard Burnand, Isabelle Peytremann-Bridevaux

**Affiliations:** 1 Institute of Social and Preventive Medicine (IUMSP), Lausanne University Hospital, Lausanne, Switzerland

**Keywords:** Chronic disease, Disease management, Attitude, Patients, Health personnel, Qualitative research

## Abstract

**Background:**

Chronic disease management has been implemented for some time in several countries to tackle the increasing burden of chronic diseases. While Switzerland faces the same challenge, such initiatives have only emerged recently in this country. The aim of this study is to assess their feasibility, in terms of barriers, facilitators and incentives to participation.

**Methods:**

To meet our aim, we used qualitative methods involving the collection of opinions of various healthcare stakeholders, by means of 5 focus groups and 33 individual interviews. All the data were recorded and transcribed verbatim. Thematic analysis was then performed and five levels were determined to categorize the data: political, financial, organisational/ structural, professionals and patients.

**Results:**

Our results show that, at each level, stakeholders share common opinions towards the feasibility of chronic disease management in Switzerland. They mainly mention barriers linked to the federalist political organization as well as to financing such programs. They also envision difficulties to motivate both patients and healthcare professionals to participate. Nevertheless, their favourable attitudes towards chronic disease management as well as the fact that they are convinced that Switzerland possesses all the resources (financial, structural and human) to develop such programs constitute important facilitators. The implementation of quality and financial incentives could also foster the participation of the actors.

**Conclusions:**

Even if healthcare stakeholders do not have the same role and interest regarding chronic diseases, they express similar opinions on the development of chronic disease management in Switzerland. Their overall positive attitude shows that it could be further implemented if political, financial and organisational barriers are overcome and if incentives are found to face the scepticism and non-motivation of some stakeholders.

## Background

Chronic diseases are increasing worldwide and are projected to be the leading causes of deaths and disability in 2030 [1]. To tackle this burden, healthcare systems have begun to rethink the organization of chronic care, and several countries have developed chronic disease management (CDM) initiatives centred on patients’ needs and based on formal evidence of effectiveness [2-5]. These initiatives aim at improving care of chronic diseases by encouraging healthcare providers to coordinate and integrate health services as well as to promote patients’ self-management [6].

In Switzerland interest in CDM is recent, contrary to North America and some European countries [7]. Switzerland, a federal state constituted of 26 cantons, has a healthcare system characterized by a decentralized structure where each canton decides on the organization of care [8]. Ambulatory care is mainly provided by independent private practitioners and outpatient services located in hospitals, paid on a fee-for-service basis [9,10]. In 1990, integrated care organizations like physicians’ network and Health Maintenance Organizations were created [11]. Functioning on a gate-keeping principle, these organizations reinforce the collaboration between healthcare providers and promote quality circles and use of guidelines [12]. Moreover, CDM initiatives have emerged within various settings [13,14] but remain rare. In this context, we need to understand barriers, facilitators and incentives to the implementation of CDM in Switzerland. The objective of our study was thus to explore opinions of various healthcare stakeholders on the development of CDM programs in this country. We carried out a qualitative study at national and cantonal levels to meet our aim.

## Methods

Because of the exploratory nature of the study, qualitative methods were used to collect and analyze the data. Indeed, we combined individual interviews and focus groups to explore opinions of Swiss healthcare stakeholders on CDM. Data were then treated by a thematic analysis.

### Study population

We used a purposive sampling strategy to select participants that we thought to have knowledge and experience of chronic diseases. Therefore, we defined the groups of participants we intended to interview and did not conduct individual interviews and focus groups up to data saturation.

### Individual interviews

To recruit healthcare stakeholders, we decided to contact the following organizations acting at different levels of the healthcare system:

–Ministry of health of various cantons

–Federal office of public health

–Health insurance companies

–Swiss medical association

–Swiss societies of medicine (general-internal medicine, endocrinology-diabetology, pneumology and cardiology)

–Cantonal societies of medicine

–Companies and physicians network with experience in developing chronic disease management projects

–Institute of nursing

–Swiss society of pharmacist

–Home healthcare centres

–Patients’ organizations

Each group received a letter detailing the aims of the study and asking them to give us the contact details of a key informant member interested to participate in our study. Thirty-three individual interviews, each lasting approximately one hour, were conducted: 25 in French, 8 in German.

### Focus groups

Chronic patients and medical staff were interviewed using the method of the focus groups (FGs) which allows the free debate of opinions [15]. Initially, we planned to organize FGs with patients suffering from diabetes, heart failure and chronic obstructive pulmonary disease (COPD). However, because the absence of patients’ associations targeting the latter two diseases and the lack of success of cardiologists and pneumologists in recruiting these patients, we eventually renounced to organise these two focus groups. We organized 5 FGs, each lasting approximately two hours, as following:

––One FG with diabetic patients

––Two FGs with general practitioners (GPs)

––One FG with specialists (diabetologists, pneumologists and cardiologists)

––One FG with nursing staff

To recruit diabetic patients, physicians and nurses, we contacted the association of diabetic patients and professionals association respectively. A letter was sent to members of associations; those interested by our study contacted us. Twenty-four patients agreed to participate. We selected them on the basis of their availability and on characteristics such as the place of residence, diabetes type, age or gender. Ten patients finally participated to our FG. Sixty seven professionals contacted us and were selected on the basis of place and number of years of practice. Finally, 23 professionals participated in our FGs. Patients and professionals received a letter detailing the aims of the study, date, time and location of the FG.

All participants were assured that the data would be confidential and anonymous. Approval was received from the Ethics Committee on Research on the Human Being of the canton of Vaud.

### Data collection

An interview guide was developed and pilot-tested. It consisted of open-ended questions on the following main topics:

–Experience and quality of chronic care

–Means to improve chronic care

–Opinions on CDM programs

–Barriers and facilitators to the development of CDM programs, incentives for participation.

Before exploring their opinions on CDM, we first asked participants if they had previously heard about it. If they did not know CDM, we briefly defined it. Indeed, we explained that it was a means to eliminate care fragmentation, that it included patients’ self-management and professionals’ teamwork, and that CDM programs were based on formal evidence of effectiveness and were adapted to patients’ needs. The latter point was illustrated with a figure of Kaiser’s triangle. For the FGs, we also added a clinical vignette presenting the story of a fictive patient benefiting from CDM.

The present study focuses only on the barriers, facilitators and incentives to the development of chronic disease management programs.

Two researchers, specialized in qualitative research methods, conducted the individual interviews and the FGs between October 2009 and June 2010. All individual interviews and FGs were audio-taped and transcribed verbatim.

### Data analysis

Analysis was carried out inductively, because of the exploratory nature of the study. Thematic analysis, used in content analysis, was chosen to reduce the content of discourses without loss or distortion of information. Thematic analysis is also an appropriate method for exploring opinions by “identifying, analyzing and reporting patterns (themes) within data” [16]. Transcripts were first analyzed line by line and divided into sequences representing themes (thematic sequences). Thematic sequences about barriers, facilitators and incentives to participation were then identified. They were finally classified according to several levels: political, financial, organizational and structural, and professionals or patients.

One researcher (SL) coded all transcripts. Checks of transcripts codes and information exchange with the last author (IPB) were regularly conducted so as to ensure consistency.

### Presentation of the results

Results have been presented separately for each classification level, always presenting barriers first, then facilitators and incentives (when appropriate). We will refer to barriers when people mentioned an existing element that could impede CDM programs. Facilitators will refer to an element that could either already exist or could be implemented to facilitate CDM programs. Incentives refer to elements that could motivate actors to participate to a program. Barriers, facilitators and incentives could either be mentioned explicitly (in answer to our questions) or implicitly, meaning that we considered the discourse of our participants as possible barriers, facilitators and incentives. The implicit elements will always be specified and presented after those which are explicit. For each result, we mentioned which group(s) of participants was/were the source. They were categorized as follows:

a) Patients (patients’ FG and the individual interview with a representative of patients organization)

b) Physicians (FGs with GPs and specialists as well as the individual interviews with members of : the Swiss medical association, Swiss societies of medicine and cantonal societies of medicine)

c) Nursing staff (nurses’ FG and the individual interviews with nurses manager and administrators of home healthcare)

d) Pharmacists (individual interview with a member of the Swiss society of pharmacists)

e) Representatives of departments of public health (DPH) (individual interviews with ministries of health and with representatives of the federal office of public health)

f) Health insurers (individual interviews with health insurers)

g) Companies proposing chronic disease management programs (interviews with administrators of companies and physicians’ networks that have developed such projects)

## Results

Generally, our results show that despite their different positions and roles in the healthcare system, the stakeholders mentioned similar barriers, facilitators and incentives to the development of CDM in Switzerland.

### Political level

Except for patients, all stakeholders mentioned the federalist political organization as a barrier to the development of CDM programs in Switzerland because it gave too much autonomy to the cantons in the organization of healthcare. Thus, CDM would be difficult to implement at a national level because each canton functions differently.

### Extract

“(…)the problem is that our sanitary laws are cantonal (…) if they were at a federal level (…) if we said that the care is like this, it would be valid for everyone, we could do the same in the whole of Switzerland (…)but it’s not done at the federal level (…)” (pharmacist)

To overcome this barrier, all participants, except nursing staff, proposed to set up a legal framework aimed at facilitating the development of CDM. Patients, physicians, health insurers and DPH also emphasized the importance of a cantonal application of such a legal framework, to adapt CDM programs to loco-regional specificities.

The general opinion of participants on CDM (in favour or not) could also be viewed as either a barrier or a facilitator. Indeed some physicians, insurers and DPH were sceptical about the necessity of implementing CDM in Switzerland, for three reasons. Firstly, they mentioned that CDM had been implemented in countries where it was necessary to change healthcare system because of a lack of healthcare structures. Conversely, Switzerland possessed many local healthcare structures and did not need to change its system.

### Extract

“(…) In the United-States, contrary to Switzerland, there are lots of patients who are coached for distance reasons, because they don’t have a hospital on each street corner like us, in Switzerland we rather have an excessive offer (…)” (Health insurer)

Secondly, they specified that care in Switzerland was already adapted to patients’ needs and disease severity, as proposed in CDM programs. Thirdly, they mentioned that work in multidisciplinary teams already occurred in informal network (that is, the personal network of the physician). Thus, CDM was only seen as a means to formalize what already exists.

At the same time, all participants were in favour of CDM considering that Switzerland possessed all the necessary resources for its implementation. The groups of physicians, nursing staff, DPH and companies proposing CDM programs noted that the main actors were aware of the problems of current chronic care. They mentioned the development of several initiatives aiming to improve chronic care, even if they occurred at a loco-regional level and had been more broadly developed in the German part of Switzerland.

At the political level, we found no themes that we could consider as incentives. Results were summarized in Table 1.

**Table 1:** Political barriers and facilitators to CDM development by group of participants

		**Patients**	**Physicians**	**Nursing staff**	**Pharmacists**	**Health insurers**	**DPH**	**Companies proposing CDM prog.**
**Barriers**	Federalist political organisation		x	x	x	x	x	x
	Scepticism about the necessity of CDM		x			x	x	
**Facilitators**	Legal framework to CDM development	x	x		x	x	x	x
	Loco-regional application of the framework	x	x			x	x	
	Favorable opinions towards CDM	x	x	x	x	x	x	x
	Availability of existing resources	x	x	x	x	x	x	x
	Awareness of actors towards current chronic care problems (with development of CDM initiatives)		x	x			x	x

### Financial level

All participants mentioned financial barriers. They raised the problem of who can pay for CDM because some components (such as prevention, self-management education or coordination for teamwork) are currently not easily reimbursed by health insurance companies.

**Table 2:** Financial barriers, facilitators and incentives to CDM development by group of participants

		**Patients**	**Physicians**	**Nursing staff**	**Pharmacists**	**Health insurers**	**DPH**	**Companies proposing CDM prog.**
**Barriers**	Who can pay for CDM	x	x	x	x	x	x	x
	Costs increasing with CDM development		x	x				
	Cost decreasing in HMO due to risk selection				x		x	
	Lack of motivation of health insurer to reimburse CDM	x					x	x
**Facilitators**	Proofs of cost-effectiveness of CDM		x	x		x	x	x
	Reimbursement of CDM components by health insurance companies adopting long term vision	x	x			x	x	
	Costs decreasing with CDM development	x	x				x	
	Cost stability		x	x		x		x
**Incentive**	Better risk compensation scheme to motivate health insurances to reimburse CDM	x					x	x

### Extract

“(…)To try to find possibilities to reimburse coordination services of (…) that is very difficult, it doesn’t fit in the reimbursement system (…), it’s very difficult to find solutions for these coordination services which are not medical services (…)how can we justify that we pay for something which is in the background?(…)” (DPH)

As facilitator to CDM development, all stakeholders, except patients and pharmacists, wanted to have proofs that CDM is cost effective. Moreover, health insurers should adopt a long term vision and reimburse programs (according to patients, physicians, health insurers, DPH).

Nevertheless, patients, DPH and companies proposing CDM programs estimated that it would be difficult to motivate health insurers to reimburse components of CDM programs since they are not integrated in the basic health insurance package. Moreover, the present risk compensation scheme encourages health insurance companies to select low risk patients rather than attract patients with chronic diseases. To avoid this problem and create incentive for health insurers, improvement of the Swiss health insurance compensation scheme was proposed.

### Extract

(Speaking about what could be done to motivate health insurance companies to participate in CDM) “ (…) It's modifying risk compensation so that health insurances are interested to do this kind of thing, that is, are also interested to have chronic patients or more expensive patients with them (…)" (Patients)

Other answers of our participants could be also viewed as barriers as well as facilitators. Patients, physicians, nursing staff, DPH and companies proposing CDM programs disagreed on the economic impact of CDM programs. While some considered that CDM would reduce health costs (by decreasing the number of hospitalization thanks to prevention activities), others imagined that CDM would increase health costs because of costs related to the implementation process of CDM programs, and to increasing patients’ life expectancy. Finally, some did not expect changes in healthcare costs, and others (pharmacist and DPH) mentioned that cost reductions observed in Swiss HMO could be explained by risk selection. Results were summarized in Table 2.

### Organizational and structural levels

Nursing staff and DPH cited the fear of transparency as a barrier, because institutions did not want to publicly show their data.

### Extract

“(…) When we want to show the outcomes (…) everyone is afraid (…) and here there are certainly barriers (…) we don’t want to be transparent (…) there is a deep anxiety to show how it goes here (…) we have to do an evaluation, we know that when we have the financing in one hand (…) and in the other hand a transparency in outcomes, things change very quickly (…)” (Nurses)

The lack of statistics on chronic disease patients was also raised by physicians and DPH as a barrier to the development of CDM.

The stakeholders did not mention solutions to overcome each of these barriers. They nevertheless evoked some facilitators at the organizational and structural levels’. Indeed, according to patients, physicians, nursing staff and DPH, CDM should be implemented within existing care structures, such as home healthcare or patients’ association, because they shared common program elements (teamwork for example).

The nursing staff added that CDM should be managed independently by healthcare professionals and not with an economic perspective by health insurers. Physicians, nursing staff and DPH also expressed the need to clearly define the stages and functioning of CDM programs.

At the organizational and structural levels, the participants’ positive attitude towards CDM can be considered as a facilitator to CDM development. Indeed, some described CDM as a means to improve quality of care through better comprehensive care as well as a means to favour communication between hospitals and ambulatory care services. Also all participants except nursing staff and pharmacists considered that CDM would encourage, within care organizations, the setting up of electronic medical records or phone consultations, as well as the use of guidelines.

### Extract

“(…) so it means (…) to change the services that are provided (…) I mean we know the best practices in diabetics' care, we have them, why are they not implemented? (…)" (DPH)

No incentives were mentioned at the organizational and structural levels. Results were summarized in Table 3.

**Table 3:** Organizational and structural barriers and facilitators to CDM development by group of participants

		**Patients**	**Physicians**	**Nursing staff**	**Pharmacists**	**Health insurers**	**DPH**	**Companies proposing CDM prog.**
**Barriers**	Fear of data transparency			x			x	
	Lack of statistics on chronic disease patients		x				x	
**Facilitators**	Development of CDM on the basis of existing structure	x	x	x			x	
	Independent management of CDM by health care professionals			x				
	Clear stages and functioning of CDM		x	x			x	
	Positive attitude to CDM	x	x	x	x	x	x	x
	Comprehensive care with CDM		x	x		x		x
	Improved coordination between hospital and ambulatory care		x			x	x	
	Setting of tools and use of guidelines within CDM	x	x			x	x	x

### Professionals’ level

Physicians, nursing staff and DPH mentioned that the supposed and feared rigidity of the CDM programs would curtail the current autonomy and freedom of practice. The loss of autonomy was considered as a barrier to CDM development. These participants, with companies proposing CDM programs, added the barrier of the “corporatism” which led to the “compartmentalization” of disciplines, and thus, impeded collaboration. All participants, except pharmacists and DPH, also mentioned the barrier of the shortage of physicians as well as the problem of the ratio of general practitioners/specialists in rural regions. Finally stakeholders, except patients and physicians, also noted actors’ inertia and lack of interest leading to slow decisions and reluctance to change.

As facilitators, pharmacists, DPH and companies proposing CDM programs expressed the need of a change in mentality and culture. According to nursing staff, insurers, DPH and companies proposing CDM programs, a common will of all the actors involved in care would also be necessary.

### Extract

“(…) we have all the infrastructure to do anything (…) but it has to become the custom, the practise (…) it has to reach everyone, all the actors and patients and it will certainly take time (…) everything take time here (laugh) it needs a real willpower (…) not only politic, of all the actors (…)” (Health insurer)

To facilitate the coordination of all professionals involved in care, physicians, health insurers, DPH and nursing staff, emphasized that the role of each professional should be defined. All the participants except patients and pharmacists also expressed the need to designate a care coordinator. According to physicians, health insurers and DPH, this coordinator should ideally be the GP. In any case, the healthcare professionals participating in CDM program should be trained to the functioning of CDM programs and to work in team. However, almost all participants considered that it would be difficult to motivate physicians to participate in CDM, especially older GPs who are used to work independently.

### Extract

“(…) physicians are individualist maniacs, physicians alone in their surgery since 25 years who are over 55 years old, it will be difficult to integrate them in such systems (…)" (Physicians)

To motivate healthcare professionals to participate in CDM programs, three types of incentives were proposed. The first one, quality incentives, included several conditions: the certainty that CDM improves quality of care, the possibility for physicians to have a help with their work load (thanks to the task delegation), and to have practical experience of a CDM program as well as to participate in its implementation and development. Teamwork and quality circles could also motivate professionals by breaking loneliness and giving the opportunity to exchange experience. The second incentive, financial, was extensively discussed by participants. Indeed they evoked this form of incentives even if some feared that it could only motivate people for economic reasons. However, in any case, participants said that quality incentives should prevail over financial incentives. The third incentive aimed at resolving the problem of financial incentives by integrating CDM components into the services usually provided by healthcare professionals (according to physicians, nursing staff and DPH). Results were summarized in Table 4.

**Table 4:** Barriers, facilitators and incentives to CDM development by group of participants at the professionals’ level

		**Patients**	**Physicians**	**Nursing staff**	**Pharmacists**	**Health insurers**	**DPH**	**Companies proposing CDM prog.**
**Barriers**	Lack of autonomy and freedom of practise with CDM		x	x			x	
	Corporatism		x	x			x	x
	Shortage of physicians and inappropriate GP/specialists ratio	x	x	x		x		x
	Inertia with lack of interest			x	x	x	x	x
	GP’s lack of motivation		x	x		x	x	x
**Facilitators**	Mentality and culture change to accept CDM				x		x	x
	Common will			x		x	x	x
	Definition of the role of each professionals’		x	x		x	x	
	Designation of a care coordinator		x	x		x	x	x
	GP as the care coordinator		x			x	x	
	Availability of CDM training	x	x	x			x	x
**Incentives**	Quality incentives	x	x	x	x	x	x	x
	Financial incentives	x	x	x	x	x	x	x
	Integration of CDM into services usually reimbursed		x	x			x	

### Patients’ level

Participants did not mention barriers at this level, expecting that patients would favour CDM. They also mentioned the program’s adaptation to patients’ needs as a facilitator to CDM development.

### Extract

“(…) The program would be an assessment, first we see where the patient is, where potentials are (…) and afterwards (there is) an education targeted on gaps (…) so that's very important, first an assessment and then to come to an agreement with the patient on what he wants to change (…)" (companies proposing CDM programs)

Participants, except companies proposing CDM programs, also proposed to reinforce patients’ own responsibility in their care. An increased supply of self-management classes were thus proposed by all participants except pharmacists. Physicians and companies proposing CDM programs emphasized the importance of checking that patients understood correctly the information received.

However, physicians, nursing staff and companies proposing CDM programs considered that it would be difficult to change patients’ habits. To motivate patients, our participants suggested quality incentives such as CDM information, awareness of CDM benefits and assurance that patients would always be in touch with the same professionals. The possibility to integrate a support group was also mentioned. All participants, except patients and pharmacists, also evoked financial incentives. Indeed, nursing staff and DPH found that the financial aspects of care were important for patients. By contrast, physicians and health insurers expressed their scepticism about the effectiveness of such incentives because, according to them, they could lead to patients’ participation to CDM for economic reasons, or to a “two-speed healthcare”, with some patients preferring to pay more for having free choice of care.

### Extract

“(…) Financial incentives raise social problems because (…) if we propose a discount to someone that earns a lot of money, he doesn’t care, but if you propose this discount to someone who doesn’t earn a lot of money and who has children, it’s not an option anymore, it becomes an obligatory choice, and thus, the free choice is emptied of its sense (…)” (physicians)

Results were summarized in Table 5.

**Table 5:** Barriers, facilitators and incentives to the development of CDM by group of participants at the patients’ level

		**Patients**	**Physicians**	**Nursing staff**	**Pharmacists**	**Health insurers**	**DPH**	**Companies proposing CDM**
**Barrier**	Difficulties to change patients’ habits		x	x				x
**Facilitators**	Supposition of a favourable opinion of patients on CDM		x			x	x	
	Adaptation of CDM to patients needs	x	x	x	x	x	x	x
	Reinforcement of patients’ responsibility in care	x	x	x	x	x	x	
	More self-management education classes	x	x	x		x	x	x
	Check the patients’ understanding of information		x					x
**Incentives**	Quality incentives	x	x	x	x	x	x	x
	Financial incentives		x	x		x	x	x

## Discussion

Our results show that the various healthcare stakeholders mentioned similar barriers, facilitators and incentives to the development of chronic disease management (CDM) in Switzerland at a political, financial, organizational-structural, professionals and patients level.

Given that the main identified barrier to CDM development is the problem of the federalist organization of Switzerland, it would be difficult to implement CDM nationally, as did some countries with national healthcare systems. Indeed the literature shows that national decisions facilitate the development of CDM [4]. CDM initiatives were thus implemented at national levels in the UK (for instance, community matrons for older complex chronic people) and more recently at a very large scale, in Germany. In this country, since 2003, health insurers have been encouraged to develop CDM programs, and incentives were created to facilitate the participation of patients and primary care physicians [17]. However, CDM has also been developed for several years at regional levels, in countries such as the Netherlands, the USA and Canada, and more recently in others in Europe [4]. A federalist political organization could also be viewed as a chance to implement CDM programs adapted to loco-regional specificities. This explains why our participants recommended a cantonal application of the legal framework.

The second barrier mostly evoked by our participants, and often cited by authors is the problem of who could pay for CDM [18], because its components are assimilated to management, coordination and prevention activities currently not reimbursed. In fact, our participants considered that the cost-effectiveness of CDM needs to be proved to convince health insurers to reimburse CDM and healthcare stakeholders of its utility. Indeed, our participants were themselves not convinced by the cost-effectiveness of CDM. Studies have shown that cost saving depends on the disease targeted, the institution where CDM is implemented and the mode of payment, so clear evidence on costs reduction with CDM are currently lacking [19-21].

Finally, our participants often tackled the issue of patients’ and professionals’ participation to CDM, and of participation incentives. Others have shown that quality incentives could encourage healthcare professionals to participate [3] in particular the conviction that CDM improves quality of care [22] or that teamwork, a component of CDM, is beneficial [23]. Our participants raised doubts about the efficacy of financial incentives, which have been shown to have limited impact especially on the physicians’ participation to CDM [24]. There are unfortunately few studies that explored opinions on patients’ motivation and incentives for their participation to CDM or to one component of CDM [25].

The stakeholders interviewed did not mention the importance of leadership as a facilitator to CDM. This contrasts with other studies [26-28] exploring this issue within institutions where CDM had already been implemented. In fact, the only stakeholder that has spoken about leadership in our study was one of the participants who had implemented CDM in his organization.

The strength of our study is that we provided the opinions of various healthcare stakeholders on the development of CDM. Therefore, this multiplicity of points of views represents a great opportunity to better understand barriers, facilitators and incentives to CDM. This should be of interest both for Switzerland and other countries facing the challenge of improving care for chronic diseases, especially since opinions converged in several ways. Two limitations of this study need to be considered. First, some participants were not familiar with CDM and may have misunderstood what it represented, despite preliminary explanations during the interview. Since CDM development in Switzerland is in its infancy, some participants may have had difficulties to imagine the practical developments of something which was, for them, too theoretical. Second, we interviewed stakeholders of some Swiss regions, thus our results cannot allow generalization to all Swiss healthcare stakeholders. However, the aim of a purposive sampling strategy is to select people with various characteristics relevant to the study objective. Qualitative research also aims to explain in details the phenomenom studied, not to provide representative results.

## Conclusions

Despite their different role and interest regarding chronic diseases, healthcare stakeholders suggested similar barriers, facilitators and incentives to the development of CDM programs in Switzerland. These common opinions and their overall positive attitude towards CDM suggest that it could be further implemented if political, financial and organizational barriers as well as the scepticism and non-motivation of some stakeholders were overcome.

## Competing interests

The authors declare that they have no competing interests.

## Authors’ contributions

SL helped to design the study, conducted most of the interviews and FGs, analysed and interpreted data and drafted paper. BB contributed to the writing of the manuscript by providing critical revision: IPB conceived, designed and supervised the study, and contributed to the writing of the manuscript. SL and IPB reached consensus regarding the data’s coding. All authors read and approved the final manuscript.

## Pre-publication history

The pre-publication history for this paper can be accessed here:

http://www.biomedcentral.com/1472-6963/12/176/prepub
